# Design and evaluation of a low-cost instrumented glove for hand function assessment

**DOI:** 10.1186/1743-0003-9-2

**Published:** 2012-01-17

**Authors:** Ninja P Oess, Johann Wanek, Armin Curt

**Affiliations:** 1 SCI Research Center, University of Zurich, Balgrist University Hospital, Forchstrasse 340, 8008 Zurich, Switzerland

## Abstract

**Background:**

The evaluation of hand function impairment following a neurological disorder (stroke and cervical spinal cord injury) requires sensitive, reliable and clinically meaningful assessment tools. Clinical performance measures of hand function mainly focus on the accomplishment of activities of daily living (ADL), typically rather complex tasks assessed by a gross ordinal rating; while the motor performance (i.e. kinematics) is less detailed. The goal of this study was to develop a low-cost instrumented glove to capture details in grasping, feasible for the assessment of hand function in clinical practice and rehabilitation settings.

**Methods:**

Different sensor types were tested for output signal stability over time by measuring the signal drift of their step responses. A system that converted sensor output voltages into angles based on pre-measured curves was implemented. Furthermore, the voltage supply of each sensor signal conditioning circuit was increased to enhance the sensor resolution. The repeatability of finger bending trajectories, recorded during the performance of three ADL-based tasks, was established using the intraclass correlation coefficient (ICC). Moreover, the accuracy of the glove was evaluated by determining the agreement between angles measured with the embedded sensors and angles measured by traditional goniometry. In addition, the feasibility of the glove was tested in four patients with a pathological hand function caused by a cervical spinal cord injury (cSCI).

**Results:**

A sensor type that displayed a stable output signal over time was identified, and a high sensor resolution of 0.5° was obtained. The evaluation of the glove's reliability yielded high ICC values (0.84 to 0.92) with an accuracy error of about ± 5°. Feasibility testing revealed that the glove was sensitive to distinguish different levels of hand function impairment in cSCI patients.

**Conclusions:**

The device satisfied the desired system requirements in terms of low cost, stable sensor signal over time, full finger-flexion range of motion tracking and capability to monitor all three joints of one finger. The developed rapid calibration system for easy use (high feasibility) and excellent psychometric properties (i.e. reliability and validity) qualify the device for the assessment of hand function in clinical practice and rehabilitation settings.

## Background

Hand function impairment caused by a neurological disorder such as stroke and cervical spinal cord injury (cSCI) has a high impact on the independence and quality of life of the affected person. Physical training therapy is of high clinical importance to improve motor recovery. Therefore, considerable efforts have been directed towards the development of new upper limb function rehabilitation therapies based on robots [1-3], passive workstations [4,5] and functional electrical stimulation (FES) systems [6,7]. Nevertheless, the overall clinical value of these therapies and a thorough evaluation of their specific advantages and disadvantages against conventional and competitive novel approaches needs to be established.

Traditional clinical upper limb function assessment tests, such as the Action Research Arm Test (ARAT) [8] and the Wolf Motor Function Test (WMFT) [9], mainly focus on the accomplishment of activities of daily living (ADL)-based tasks, while the motor performance, i.e. kinematics, is less detailed. However, these details might allow not only for the distinguishing of different patterns of impairment but also for a close following of the course of hand function during treatment. In addition, traditional tests are mainly based on ordinal rating, and are as a result subjective and somewhat imprecise. Thus, an objective and precise tool is required for hand motor performance evaluation.

Dynamic recordings of finger bending angles during the performance of skilled tasks, such as grasping objects, can be performed with 3D motion capture systems and with instrumented gloves. The 3D motion capture systems have the disadvantage of being bulky and are less suitable for routine clinical use. Since the late seventies, numerous instrumented gloves have been developed in industry and academia for applications in fields such as computer gaming, virtual reality, sign language recognition, medicine, rehabilitation and robotics. As described by Dipietro et al. [10], different sensing technologies have been used to design these gloves. Resistive bend sensors represent a lightweight and inexpensive alternative [11]; therefore, most gloves currently integrate this sensing technology. Indeed, bend sensors cost approximately $7 per unit, and are thus the most inexpensive technology for designing instrumented gloves.

The main bend-sensor-based gloves are: the Wü-Glove [12], the Shadow Monitor [11,13], the DG5 VHand (DGTech Engineering Solutions, Bazzano, Italy), the Cyberglove II (Virtual Technologies Inc., Palo Alto, CA), the BabyGlove [14], the P5 Glove (Essential Reality Inc., Mineola, NY) and the Sigma Glove [15]. These gloves present drawbacks, such as sensor signal drift, sensor saturation, the inability to monitor the three joints of one finger and/or a time-consuming calibration procedure. Only few of them have been assessed for reliability, validity and/or feasibility, and none of them became routinely used for the evaluation of hand function impairment in clinical practice.

The goal of this study was to develop a low-cost instrumented glove to capture finger bending and provide a feasible tool to assess hand function in clinical practice and rehabilitation settings. Important requirements for the glove design were sensor signal stability over time, the ability to monitor the full range of motion in finger-joint bending and the capacity to capture the bending of all three joints of one finger. In addition, a rapid calibration system and a high resolution were also essential. Moreover, the glove had to be tested for reliability, validity and feasibility. The design of the glove, termed the NeuroAssess Glove, is presented, and the reliability, validity and feasibility evaluations are described.

## Methods

### Sensor selection

Resistive bend sensors have a finger-joint flexion angle measurement domain that ranges between 0° and approximately 100°. We tested gloves (the P5 Glove and the DG5 VHand) that incorporate a single 4.5-in long sensor per finger and observed that the sensors saturated as soon as the sum of the bending angles of the three joints of one finger (two for the thumb) reached approximately 100°. In consequence, to avoid saturation, we decided to cover each finger joint that we planned to monitor with an individual sensor.

According to Simone and Kamper [11], resistive bend sensors from Flexpoint Sensor Systems (Draper, UT) [16] present the greatest signal stability over time in comparison to sensors from Abrams Gentile Entertainment, Inc. (New York, NY) [17] and Spectra Symbol (Salt Lake City, UT). Among the different sensor sizes commercially available from Flexpoint (4.5 in, 3 in, 2 in and 1 in), we determined that the appropriate sensor size for covering a finger joint was 2 in. To establish whether the size of a sensor had an impact on its output signal stability over time, we evaluated the step responses of 4.5-in, 3-in and 2-in bare sensors from Flexpoint as well as of 4.5-in sensors from Abrams Gentile for comparison.

The sensors' signal stability was assessed in a manner similar to that reported by Simone and Kamper [11]. Seven sensors of each type were tested. Each sensor was initially fixed at its proximal end on a 3-in diameter cylinder for several seconds, then bent over the cylinder for at least 30 s and finally unfolded again. The sensor's step response was recorded and the percentage of drift (i.e., the ratio between the initial and final voltage amplitude difference, and the initial voltage amplitude in percentage) was determined after 30 seconds. For one sensor of each type, the measurement was repeated five times and the percentage of drift was calculated from the average of the five measurements.

### Glove design

The NeuroAssess Glove was fabricated for the right hand; it was made of 0.38 mm thick polyamide/Lycra stretchable functional tulle (Ref. PN 171, Liebaert n.v., Belgium). This fabric was chosen due to its broad stitches, which allow more contact between the skin and the grasped object compared to other types of materials. The glove prototype exists in three different sizes: small, medium and large.

**Figure 1:**
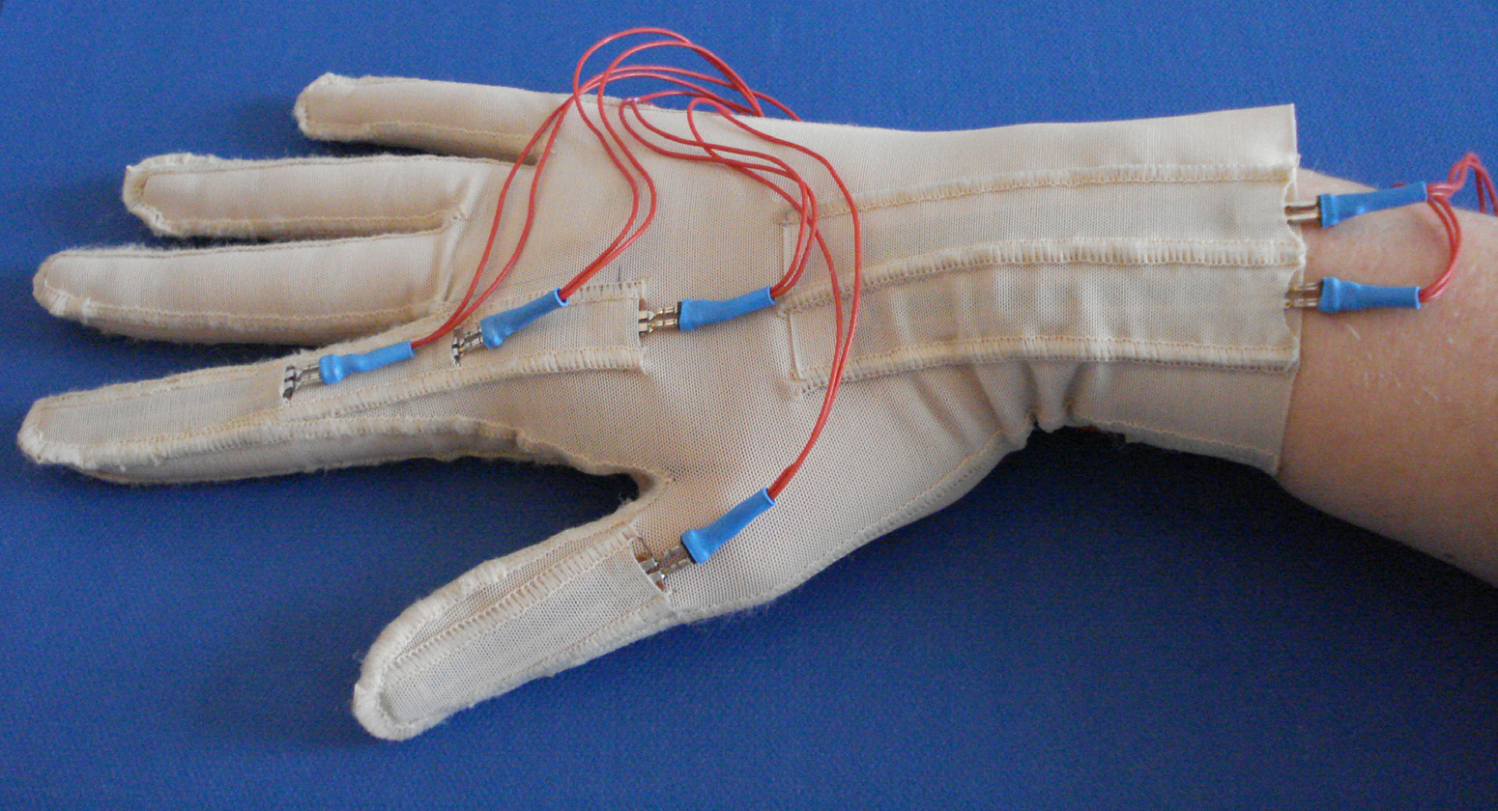
**Top side view of the NeuroAssess Glove**. The glove has integrated sleeves into which the sensors were inserted. Four 2-in sensors were used to monitor finger bending of the index MCP, PIP, DIP and thumb IP joints, and two 3-in sensors were used to capture palmar and dorsal flexion of the wrist. The sensors' proximal ends were fixed to the glove with medical tape to avoid displacement during finger motion monitoring. For the sake of clarity, the medical tape is not shown in this figure.

The glove was equipped with polyester over-laminated resistive sensors from Flexpoint. As shown in Figure 1, the glove has integrated sleeves into which the sensors were inserted. Four 2-in sensors were used to cover the index metacarpophalangeal (MCP), proximal interphalangeal (PIP), distal interphalangeal (DIP) and thumb interphalangeal (IP) joints. We decided to limit finger flexion and extension monitoring to these finger joints given that we observed that they displayed the greatest changes in bending angles during the performance of the main grasp forms. Nevertheless, we do not exclude the additional capturing of movements of the ring, middle and little fingers as well as of the MCP joint of the thumb in a further development step of the glove. Moreover, two 3-in sensors covered the radiocarpal joint to determine whether patients made compensatory movements with the wrist. One sensor monitored palmar flexion, whereas the other one monitored dorsal flexion of the wrist. However, in this study, the focus was placed on the analysis of finger-joint bending rather than on wrist bending.

The sensors were placed at their middle length over the finger joints. Their proximal ends were fixed to the glove fabric with medical tape to avoid displacement during finger motion monitoring. Depending on the length of the subject's finger, the distal end of a sensor could overlap the proximal end of another one. To avoid contact between sensors placed on the index, three different layers of polyamide/Lycra fabric were sewn on the glove fabric, thereby forming separate sleeves into which the sensors were inserted.

Each sensor cable was connected to its physically separated signal conditioning unit. The conditioning unit was wired to an 8-channel, 12-bit, 10kS/s NI-6008 analog digital converter (National Instruments Corp., Austin, TX) and the converter was plugged into a portable computer via a USB cable. The sensor signals were sampled continuously at 100 Hz using LabVIEW (NI Corp., Austin, TX).

### Voltage-to-angle conversion

The conversion from sensor output voltages into angles (in degrees) is usually performed by means of a calibration procedure before each measurement session. In this procedure, a traditional goniometer is used to measure a few finger bending angles, and the corresponding sensor output voltages are determined. From these voltage-angle pairs, a nonlinear [13,15] or linear [12] relationship is obtained by interpolation, from which output voltages can be converted into angles. Such a calibration procedure is time-consuming, tedious and a source of inaccuracy [10].

The NeuroAssess Glove was based on a different conversion system, wherein the relationship between the sensors' output voltages and finger bending angles was pre-measured using an automated instrument. The instrument contained a dummy finger part, whose joint could rotate with 0.5° steps in a 0°-120° range. The sensors were placed over the dummy finger joint, then bent over the joint from 0° to 120° and finally unfolded from 120° to 0°. For each sensor, three voltage-versus-angle curves were pre-measured. Their average was stored in a lookup table, which enabled a program to convert the output voltages read from the sensor into angles. To take into account the sensor hysteresis, only the curve measured when the sensor was unfolded was considered. Furthermore, to ensure the maximal accuracy for the sensors embedded in the glove, these sensors were placed at the same position (that is, at their middle length) on the dummy finger joint as they were on the real finger joints. The advantage of this system was that the glove did not require calibration before each measurement session, which saved a significant amount of time.

### Sensor resolution enhancement

The output response of a 2-in bend sensor from Flexpoint, when bent from 0° to 120° over a finger joint, is nonlinear overall. In fact, it contains a partly linear region (from 0° to approximately 100°) and a saturation region (from approximately 100° to 120°); the latter cannot be used for measurements. Nevertheless, the shape of the sensor curve can be modified to increase the sensor dynamic range, and thereby enhance the sensor resolution, by modifying the sensor signal conditioning circuit. The sensor dynamic range refers to the interval limited by the largest and smallest measurable values.

In a previous study, we had implemented a program that was simulating a signal conditioning circuit supplied with a 5 V constant voltage, wherein the sensor was connected with a series and a parallel resistor. The simulation program showed that the parallel resistor increased the linearity of the sensor output response but considerably decreased its slope, and thereby deteriorated the sensor resolution. Furthermore, this simulation and additional validation measurements also demonstrated that a series resistor of 33 kΩ maximally enhanced the sensor resolution [18]. However, the resulting resolution of 0.5° was dependent on the longitudinal position of the sensor with respect to the finger-joint.

**Figure 2:**
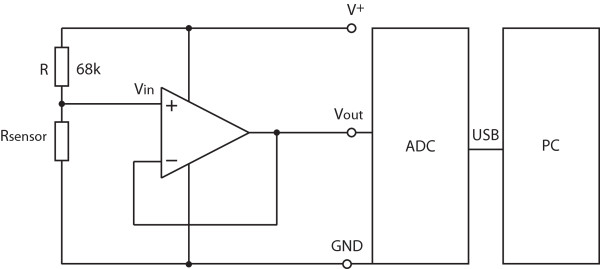
**Signal conditioning circuit of one sensor**. The circuit was supplied with a constant voltage of 10 V (V^+^) to increase the sensor dynamic range and to enhance the sensor resolution. The circuit was modified appropriately, whereby, the sensor was connected in series with a 68-kΩ resistor (R), and a non-inverting impedance converter was implemented between the circuit and the analog digital converter (ADC).

Our goal in this study was to further enhance the sensor resolution by increasing the sensor dynamic range with a simple signal conditioning circuit. The circuit of each sensor was modified as follows: the constant voltage supply (5 V) of the initial circuit was doubled (10 V), and the circuit was modified appropriately. Specifically, the sensor was connected in series with a 68-kΩ resistor (instead of a 33-kΩ resistor) and an impedance converter was implemented between the circuit and the analog digital converter (ADC), as illustrated in Figure 2. Output responses of the sensor were measured as a function of the bending angle. For each type of circuit, three voltage-versus-angle curves were measured and their average was taken into account.

### Participants

Ten healthy subjects (4 males and 6 females, mean age 34.5 ± 12.8 years) and four inpatients (4 males, aged from 18 to 67 years) at the Balgrist University Hospital in Zurich, Switzerland, with a pathological hand function caused by a cSCI met the inclusion criteria and volunteered for the study. The inclusion criteria were as follows: 1) between 18 and 70 years old, 2) cognitive ability to follow simple verbal instructions and 3) right-handed. The study exclusion criteria were as follows: i) other neurological disease, ii) cardiovascular disease, iii) orthopedic disease and iv) mental disease. All participants received written and verbal information about the study and gave written informed consent. The protocol of the study was approved by the local ethics committee.

### Characteristics of the patients' hand function

The patients had different levels of hand function impairment. The clinicians classified and numbered the patients in a decreasing order according to their remaining hand motor functions. **Patient 1 **had a hand motor function close to that of the healthy subjects. In other words, he had voluntary control of extrinsic and intrinsic hand muscles within an entire workspace. Furthermore, he had the ability to perform different grasp forms. Yet, the sensory level of his hand was slightly reduced, which somewhat diminished his ability to grasp objects. **Patient 2 **had voluntary control of extrinsic and intrinsic hand muscles within an entire workspace. Moreover, he had the ability to perform some grasp forms. Nevertheless, his muscle strength and dexterity were reduced. **Patient 3 **had voluntary control of the wrist and some extrinsic muscles. As a result, he could perform grasping and opening and closing of the hand with or without an active tenodesis effect. The active tenodesis effect describes the passive finger extension when the wrist is flexed and, conversely, the passive finger flexion when the wrist is extended. However, his hand dexterity, strength and workspace were limited. **Patient 4 **had no voluntary control of either extrinsic or intrinsic hand muscles but could actively extend the wrist. Thus, he could generate passive finger movements by an active tenodesis effect. The grasping function was limited to a reduced workspace. **Patients 2**, **3 **and **4 **suffered from spasticity.

### Reliability evaluation

**Figure 3:**
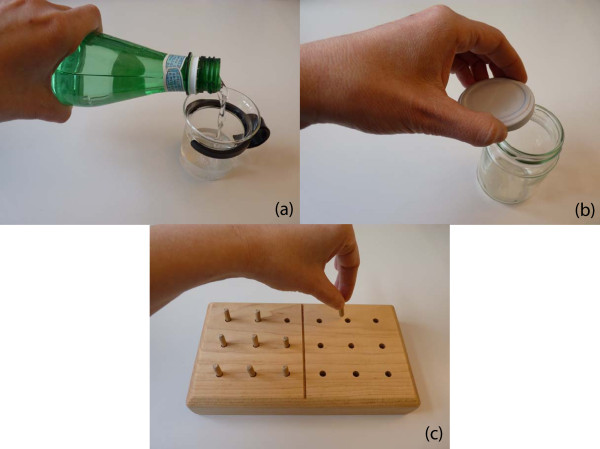
**Activities of daily living (ADL)-based tasks**. (a) Task 1. The subject had to take a bottle from the table, pour water into a glass and put the bottle back on the table. (b) Task 2. The subject was asked to unscrew the lid of a jar and put the lid on the table. (c) Task 3. The subject had to remove a peg from the hole of a pegboard and place it into the hole of another pegboard.

The reliability of the glove was assessed by determining the repeatability of the finger bending trajectories recorded from ten healthy subjects during the performance of three ADL-based tasks. In each task, a specific grasp form, identified as one of the most often used in daily functional living by Sollerman [19], had to be performed. **Task 1 **comprised the transverse volar grip; the subject had to take a bottle from the table, pour water into a glass and put the bottle back on the table (Figure 3 (a)). The spherical volar grip was included in **Task 2**; the subject had to unscrew the lid of a jar and put the lid on the table (Figure 3 (b)). The subject was allowed to use his/her left hand to stabilize the jar while unscrewing the lid. Nevertheless, the jar had to remain on the table and not be lifted into the air. **Task 3 **contained the pulp pinch; the subject had to remove a peg from the hole of a square pegboard and place it into the hole of another pegboard (Figure 3 (c)). Before and after the accomplishment of each task, the subject had to put his/her hand in a flat, neutral position on the table.

To standardize the performance of the ADL-based tasks, the position of the right hand (when in the flat, neutral position) and of the objects (the bottle, the glass, the jar and the pegboards) on the table was defined. The contour lines of the hand and of the objects were drawn on a thin synthetic non-skid desk pad, which was placed on the table in front of the subject. Furthermore, in Task 1, the amount of water inside the bottle and the level to which the glass had to be filled were also defined. In Task 3, the subject was asked to remove the peg from the top left hole of the left pegboard and to place it into the top left hole of the right pegboard. The only parameter we found difficult to standardize was the amount of screwing of the jar lid before the subject had to open it, in Task 2.

The healthy subjects had to perform each task once on two different days. Before each measurement session, the subject was helped to don the glove, and the examiner inserted the sensors into the sleeves, placed them precisely at their middle length over the finger joints, and fixed their proximal ends with medical tape on the glove fabric. For each measure, the bending trajectories of the four joints were added up and time-normalized t/Ts, where t is the time, Ts is the period for a given number of samples and s is the number of samples (s = 1000). For each task, the intraclass correlation coefficient (ICC) was calculated between the ten pairs of readings.

### Validity evaluation

The accuracy of the NeuroAssess Glove was evaluated by determining the agreement between angles measured with the sensors embedded in the glove and angles measured by traditional goniometry. Four healthy subjects, two men and two women, with one large hand size, two medium hand sizes and a small hand size, were asked to bend each finger joint 10°, 30°, 50°, 70° and 90° in a static manner. A goniometer with a 1° resolution (Protek AG, Bern, Switzerland) was placed on the dorsal aspect of the finger joint along the sensor and the angles read with both methods were compared. For each finger-joint and each angle, three measurements were performed, and the mean of the three differences was taken into account.

### Feasibility evaluation

The patients had to perform the same ADL-based tasks as the healthy subjects according to the protocol described in section "Reliability evaluation", except that they only had to perform the tasks once. The captured and processed finger bending trajectories of the patients and the average trajectories of the healthy subjects were plotted on the same graphic for pattern comparison. The first of the two measures taken in the healthy subjects for the reliability evaluation was considered.

For each task, the score of a patient was established by calculating the correlation between his curve and the curve of each of the healthy subjects using the ICC; the average of the ten values was taken into account. Similarly, the score of a healthy subject was determined by calculating the correlation between his/her curve and the curve of each of the other healthy subjects, and the average was considered. To assess whether we could differentiate between the hand function level of the patients and that of the healthy subjects with the NeuroAssess Glove, we tested the null hypothesis that the distributions of scores in the two groups were the same using the non-parametric Wilcoxon rank sum test for independent groups. Furthermore, the correlation between the scores obtained with the glove test and the classification of the clinicians was determined using the non-parametric Spearman's correlation coefficient.

In addition, the patients were asked to report any inconvenience and/or discomfort experienced with the glove during the performance of the ADL-based tasks. We did not ask any targeted questions regarding the glove's ergonomics (in contrast to previous work by others [13,12]), and we left the question open to avoid missing important information that we had not thought about.

## Results and discussion

### Sensor selection

**Figure 4:**
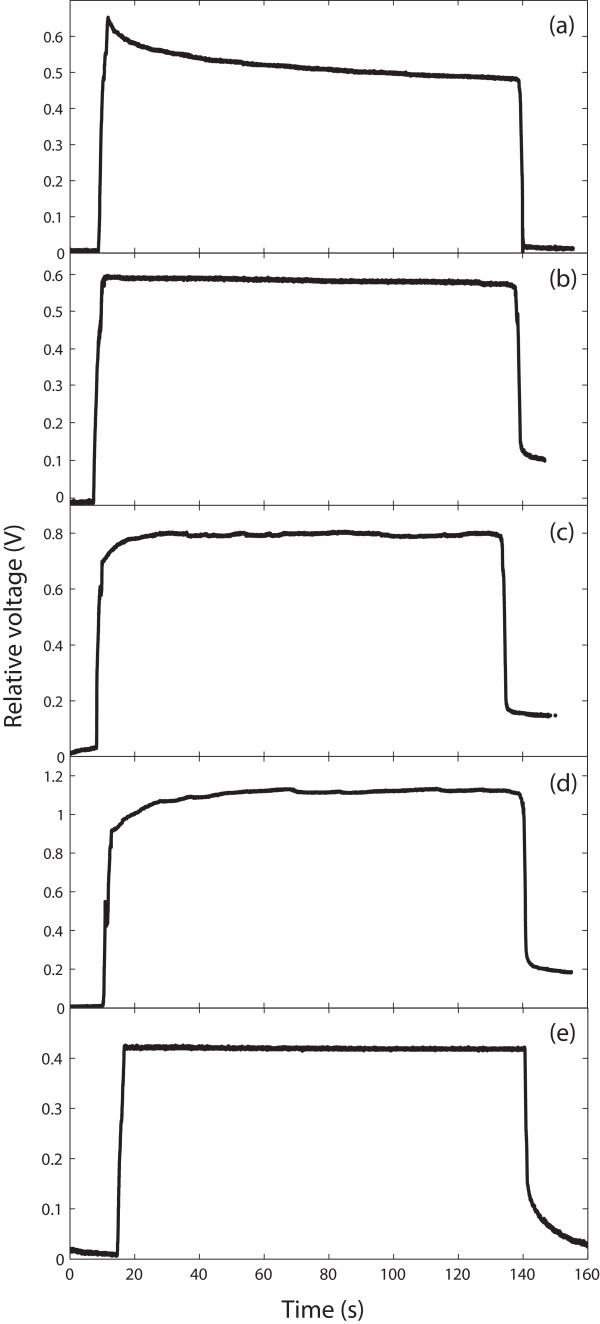
**Evaluation of sensor signal stability over time**. Step responses of (a) a 4.5-in sensor from Abrams Gentile and different sensors from Flexpoint, specifically, (b) a 4.5-in bare sensor, (c) a 3-in bare sensor, (d) a 2-in bare sensor and (e) a 2-in polyester over-laminated sensor.

The step responses of (a) a 4.5-in sensor from Abrams Gentile and bare sensors of different sizes from Flexpoint, specifically, (b) a 4.5-in sensor, (c) a 3-in sensor and (d) a 2-in sensor, are displayed in Figure 4. The corresponding signal drift percentages obtained from the average of five measurements are given in Table 1. These results are very similar to the results obtained by Simone and Kamper [11]. Indeed, we found a 22.19% signal drift in the 4.5-in sensor from Abrams Gentile, whereas they obtained 24.4%. Similarly, we found an 8.25% drift in the 3-in bare sensor from Flexpoint, whereas they obtained 8.9%. In addition, our results showed that when the sensor length decreased, the signal drift increased; the percentages of drift of the 4.5-in, 3-in and 2-in sensors were 0.9%, 8.25% and 18.20%, respectively.

**Table 1:** Sensor signal drift values

Sensor type	Sensor signal drift (%)
4.5-in sensor (Abrams Gentile)	22.19 (decrease)
4.5-in sensor, bare (Flexpoint)	0.9 (decrease)
3-in sensor, bare (Flexpoint)	8.25 (increase)
2-in sensor, bare (Flexpoint)	18.20 (increase)
2-in sensor, polyester over-laminated (Flexpoint)	0.47 (decrease)

In their study, Simone and Kamper [11] also tested sensors from Flexpoint with different configurations (over-laminated, over-molded and bare) and found that the bare sensor signal showed the lowest time-varying decay. Gentner and Classen [12] corroborated these findings, and moreover, were able to enhance the signal stability of the bare sensors from Flexpoint by fixing a thin unplasticised polyvinyl chloride (PVC) foil over the carbon layer located on the front side of the sensor.

Flexpoint typically supplies sensors with a polyimide over-laminate, a polyester over-laminate or without an over-laminate. Because the polyester over-laminate sensor configuration had not been evaluated by Simone and Kamper [11], we tested it and compared it with the bare sensor configuration. The step response of a 2-in polyester over-laminated sensor is illustrated in Figure 4 (e), and the corresponding signal drift percentage obtained from the average of five measurements is given in Table 1. The percentage of signal drift of the 2-in polyester over-laminated sensor (0.47%) was significantly lower than that of the bare sensor (18.20%). Hence, the polyester over-lamination process enhanced the sensor signal stability over time. This enhancement was comparable with that obtained by Gentner and Classen [12], which was -1.0 ± 0.8% after 40 s and -1.9 ± 1.0% after 50 min. The advantage of the 2-in polyester over-laminated sensors that we used over the same-sized laminated sensors used by Gentner and Classen [12] is that the former did not require to be modified, thereby decreasing the time of fabrication of a glove for the same cost. The sensor drift values given in Table 1 were determined from the average of five step responses recorded from a single sensor. The values obtained from different sensors of the same type did not show any substantial variability.

### Sensor resolution enhancement

**Figure 5:**
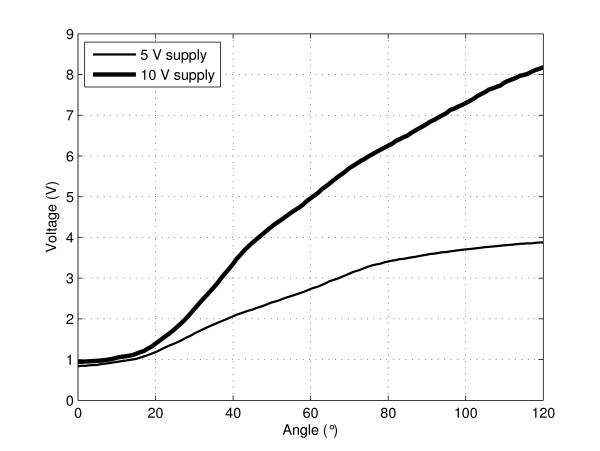
**Sensor dynamic range increase**. Sensor output responses as a function of the bending angle acquired with the signal conditioning circuit supplied with a constant voltage of 5 V and 10 V. When the voltage supply was doubled, the sensor dynamic range was more than doubled, resulting in a resolution enhancement.

Figure 5 shows the sensor output responses acquired when using a signal conditioning circuit supplied with a constant voltage of 5 V and 10 V. By doubling the amount of voltage supply, the sensor dynamic range increased from 0.8 - 3.9 V (3.1 V difference) to 0.9 - 8.2 V (7.3 V difference), and was thus more than doubled. Furthermore, sensor responses were recorded with different sensor longitudinal positions with respect to the dummy finger-joint; regardless of the position, the resolution was 0.5° over the whole 0°-120° range. This resolution was, however, limited to 0.5°, corresponding to the steps executed by the motor of the automated pre-measurement instrument.

In comparison, the sensor dynamic range of approximately 2 - 4.4 V (2.4 V difference) obtained by Simone et al. (read from Figure 5 of reference [13]) with a 3-in bare sensor sample from Flexpoint was approximately three times smaller than ours. They obtained a resolution ranging from 0.06° to 4.6°. Nevertheless, these resolution values cannot be compared to ours. The values obtained by Simone et al. [13] were extracted from curve fits and were not from measured sensor responses. Thus, their values corresponded to the resolution of their hardware and ADC settings but not to the actual sensor resolution.

In their sensor signal conditioning circuit, Gentner and Classen [12] connected a resistor in parallel with the sensor, which served as a feedback resistor of a non-inverting amplifier to increase sensor linearity and to thereby reduce calibration time. They did not report any information regarding the sensor dynamic range that they obtained with the 2-in sensors from Flexpoint that they modified. However, they communicated an overall resolution of approximately 0.1°. This value was extracted from regression lines, and, similar to the work of Simone et al.[13], it did not correspond to the actual sensor resolution.

In summary, we present an instrumented glove with a high resolution of 0.5° over the whole 0°-120° range (for any sensor position with respect to the finger-joint). The sensor dynamic range was greatly enhanced because of a simple signal conditioning circuit. However, the sensor resolution was limited by the measurement interval of 0.5° but can be further improved by changing the motor of the automated pre-measurement instrument.

### Reliability evaluation

**Figure 6:**
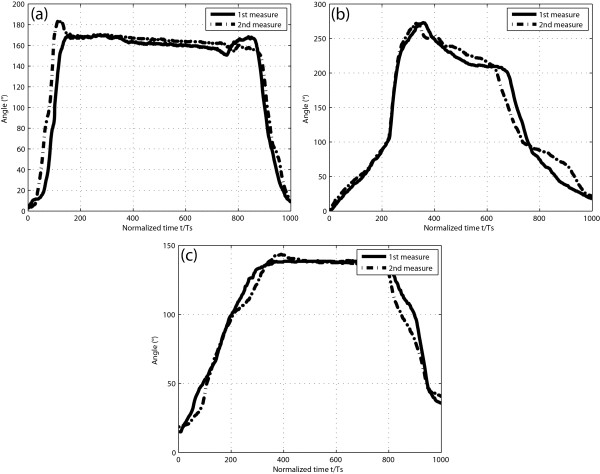
**Glove reliability evaluation**. Bending trajectories of the four finger joints (index MCP, PIP, DIP, and thumb IP joints) were added up and time-normalized following repeated performance of (a) Task 1 (subject 10), (b) Task 2 (subject 9) and (c) Task 3 (subject 7) on two different days.

The finger bending trajectories that were recorded in the ten healthy subjects on two different days and processed are displayed in Figure 6 (a) for Task 1 (subject 10), (b) for Task 2 (subject 9) and (c) for Task 3 (subject 7). A pattern specific to each task could be distinguished in the trajectories. This pattern was similar in the curves of the different subjects.

Although the subjects put their hands in the flat, neutral position before and after the accomplishment of each task, the sensor output signals were not zero at the end of the tasks. This variation was due to the fact that when the sensor was bent and then unfolded, there was a delay until the sensor response was zero. Furthermore, in some subjects with prominent finger-joints, there was an offset (bias) in the sensor output signal, as seen at the beginning of subject 7's curves, illustrated in Figure 6 (c). The mean and the range of the ICC values obtained between the 10 pairs of repeated readings are given in Table 2. A value of 0.70 or higher for a reliability coefficient is commonly a criterion of acceptability for traditional upper limb function assessment instruments [20]. Thus, the NeuroAssess Glove offers a reliable tool for recording finger motion during the performance of ADL-based tasks. Some other instrumented gloves, such as the Wü-Glove [12], the Shadow Monitor [11,13], the Humanglove [21] and the DataGlove [22], have also been assessed for reliability. Nevertheless, the evaluation involved static hand postures instead of dynamic movements.

**Table 2:** ICC values

Task n°	Mean	Range
1	0.92	0.85-0.95
2	0.91	0.81-0.98
3	0.84	0.76-0.98

We expected a lower ICC value for Task 2 (unscrew the lid of a jar) than for Task 1 (pour water into a glass) and Task 3 (remove and place a peg) given that we could not standardize the amount of screwing of the jar lid. Astonishingly, the ICC value measured for Task 3 was the lowest. This difference was not due to the length of the tasks given that the average time that it took for the subjects to accomplish Task 3 was approximately the same as for Task 2 (8.7 s for Task 1, 4 s for Task 2 and 3.6 s for Task 3). The low ICC value obtained in Task 3 might be due to the fact that either very little or no flexion of the thumb IP and/or the index DIP joints was measured, as expected with the pulp pinch. Thus, the trajectories in Task 3 corresponded to the sum of the bending angles of two, or three, finger-joints, instead of four, as in the other two tasks.

### Validity evaluation

The accuracy of the glove was assessed by comparing the angles measured with the sensors embedded in the glove with the angles measured using traditional goniometry. The mean of the observed differences (bias), the standard deviation (SD) of the differences and the 95% limits of agreement across subjects and angles are given in Table 3. The bias was -0.59, which showed that there was a small systematic difference between the pairs. Indeed, the sensor readings were mainly slightly higher than the goniometer readings. We think that a higher response was due to the fact that when a subject's finger joint was somewhat prominent, the sensor response had an offset, whereas the goniometer reading was less affected by the finger-joint thickness. The 95% limits of agreement were within ± 5°. This accuracy was similar to that of other gloves evaluated such as the Sigma Glove, the CyberGlove and the DataGlove [15]. The accuracy error of the sensor covering the thumb IP joint was greater than that of the other sensors. Our hypothesis is that some subjects had a thumb distal phalange with a rather concave geometry. The sensor part covering the thumb distal phalange followed its concave form, whereas the goniometer formed an axis joining the IP joint and the thumb tip.

**Table 3:** Agreement limits for the sensors embedded in the glove and traditional goniometry

Finger joint	Mean difference (bias)	SD of differences	95% agreement limit
Index DIP	-0.43	1.94	-4.31 to 3.44
Index PIP	-0.46	1.95	-4.36 to 3.44
Index MCP	-0.48	2.18	-4.84 to 3.89
Thumb IP	-0.98	2.59	-6.15 to 4.20
Mean	**-0.59**		**-4.91 to 3.74**

Traditional goniometry is the gold standard for clinical evaluation of finger motion. However, although these devices commonly have a 1° resolution, they are notoriously unreliable with an accuracy error estimated to be at least ± 5°. Rather than to establish the exact accuracy of the glove, the purpose of the validity evaluation was to determine if the new voltage-to-angle conversion system would generate a glove accuracy similar to that of other evaluated gloves.

### Feasibility evaluation

**Figure 7:**
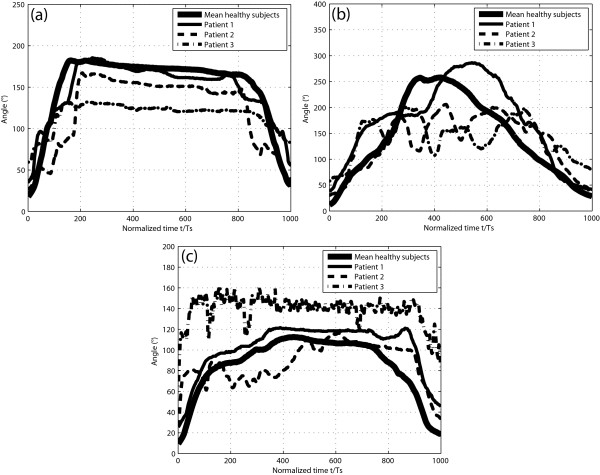
**Pattern comparison of finger bending trajectories**. Captured and processed finger bending trajectories of the patients together with the average trajectories of the healthy subjects following the performance of (a) Task 1, (b) Task 2 and (c) Task 3 are shown. The trajectories of patient 4 were not plotted on the graphics for the sake of clarity.

The captured and processed finger bending trajectories of the patients together with the average of the trajectories of the healthy subjects following the performance of the ADL-based tasks are illustrated in Figure 7 (a) for Task 1, (b) for Task 2 and (c) for Task 3. For all of the three tasks, we did not plot the curves of patient 4 on the graphics for the sake of clarity. In **Task 1**, patients 1 and 2 did not directly grip the bottle at its wider part, but instead, their semi-closed hand went along the bottle from the top narrow part down to the wider part, as seen on the lateral sides of their trajectories (especially for patient 2) in Figure 7 (a). Patients 3 and 4 could not perform the task with the bottle full. For this reason, we adapted the test for them by filling the bottle with the maximum amount of water with which they managed to accomplish the task. Patient 4 poured water into the glass with a supination instead of a pronation movement of the hand. In **Task 2**, patient 1 had to open the lid of the jar in two steps instead of the one step used by the healthy subjects. Patients 2, 3 and 4 required several steps to open the jar lid. For the latter group, the lid of the jar was closed less tightly than for patient 1 and the healthy subjects. In **Task 3**, patient 3 required several attempts before he managed to pinch the peg. Patient 4 did not succeed in pinching the peg. As a result, the examiner stopped the recording as soon as the patient reported that he could not complete the task. Consequently, the glove test could be used to assess patients with different levels of hand function impairment; nevertheless, the test had to be slightly adapted for some patients. Furthermore, it can be observed that the finger-bending trajectories of patient 3 (Figure 7) and patient 4 (not shown here) present a significantly higher offset than those of the other subjects due to the fact that they suffered from important spasticity.

The scores obtained by the participants from the NeuroAssess Glove test are given in Table 4. Across tasks, the average scores in the healthy subjects' and in the patients' groups were 0.76 and 0.45, respectively. The Wilcoxon rank sum test demonstrated that the distribution of scores in the two groups were significantly different (P < 0.01). Thus, using the glove, we could distinguish the hand function level between the two groups with great confidence. Moreover, Table 4 shows that there was a strong correlation between the mean scores obtained with the glove from the mean healthy subjects and the patients, and the classification made by the clinicians (Spearman's ρ = 1, P < 0.02).

**Table 4:** NeuroAssess Glove test scores

	Task 1	Task 2	Task 3	Mean
Mean healthy subjects	0.71	0.85	0.73	**0.76**
Patient 1	0.74	0.72	0.65	**0.70**
Patient 2	0.64	0.65	0.48	**0.59**
Patient 3	0.39	0.38	0.12	**0.30**
Patient 4	0.31	0.30	0.01	**0.21**

Regarding the user feedback, the patients did not report any reduction in sensitivity to touch, which we were afraid might happen. Instead, patients 2 and 3 mentioned a stiffness of the glove that restricted movement, and patients 3 and 4 communicated a slipperiness of the device that made prehension of an object more difficult. We believe that the stiffness was not due to the fact that the glove was too tight given that the material was stretchable and that we carefully chose the appropriate glove size for each subject's right hand. Instead, we think that the problem was rather due to the thickness of the material (0.38 mm). Given these pieces of information a thinner and non-skid material should be identified to enhance the glove's ergonomics.

## Conclusions

The design and evaluation of a low-cost instrumented glove prototype for the routine assessment of hand function in clinical practice and rehabilitation settings was presented. The glove provided a precise and objective alternative to traditional clinical hand function assessment tests. Furthermore, it focused on the hand function kinematics during the accomplishment of ADL-based tasks rather than on the ability to accomplish these tasks. The advantages of the NeuroAssess Glove in comparison to other bend-sensor-based gloves were the following: the output signals of the embedded sensors were stable over time, the full range of motion in finger-joint bending could be captured and the device could monitor all three joints of one finger (here the index). Moreover, the glove had a high resolution of 0.5° and did not require calibration before each measurement session. The device was considered to be reliable, given the high ICC values obtained (0.84 - 0.92), and was estimated to be accurate, as it displayed a ± 5° error, similar to that of other gloves evaluated. In addition, it was feasible to assess different levels of hand function damage in cSCI patients with the device. Finally, a thinner and non-skid material should be identified to enhance the glove's ergonomics.

## Competing interests

The authors declare that they have no competing interests.

## Authors' contributions

NO designed the glove, evaluated it and wrote the manuscript. JW participated in the design of the glove and in the revisions of the manuscript. AC provided feedback and expert guidance throughout this study, and contributed to the intellectual content of the manuscript. All authors read and approved the final manuscript.
